# Taxonomic study of subgenus *Plastus* s. str. (Coleoptera, Staphylinidae, Osoriinae) in China, with descriptions of five new species

**DOI:** 10.3897/zookeys.51.457

**Published:** 2010-07-23

**Authors:** Jie Wu, Hong-Zhang Zhou

**Affiliations:** 1 Key Laboratory of Zoological Systematics and Evolution, Institute of Zoology, Chinese Academy of Sciences, 1 Beichenxi Rd., Chaoyang District, Beijing 100101, P. R. China; 2 Shanghai Entomological Museum, Chinese Academy of Sciences, 300 Fenglin Rd., Xuhui District, Shanghai 200032, P. R. China

**Keywords:** Staphylinidae, Plastus s. str., new species, China

## Abstract

This paper treats Chinese species of the rove beetle genus Plastus Bernhauer, 1903, subgenus Plastus s. str. (Coleoptera, Staphylinidae, Osoriinae). None of the 87 previously described species of this subgenus are known from China. The following five new species are described and illustrated: Plastus Plastus amplus Wu & Zhou, **sp. n.** from Xizang, Plastus Plastus rhombicus Wu & Zhou, **sp. n.** from Guangxi, Plastus Plastus rhombicus Wu & Zhou, **sp. n.** from Yunnan, Plastus Plastus shanghaiensis Wu & Zhou, **sp. n.** from Shanghai, and Plastus Plastus tuberculatus Wu & Zhou, **sp. n.** from Hainan. A key to adults of the five known Chinese species is provided.

## Introduction

The subgenus Plastus s. str. Bernhauer, 1903, is a species rich rove beetle group with 87 previously recorded species (Herman 2001, Wu and Zhou 2007). Most of them are known to be closely associated with dead wood habitats in the tropical and subtropical forests of south and east Asia and the islands of South Pacific, with a few species in Madagascar (Cameron 1930, Greenslade 1971, Herman 2001, Wu and Zhou 2007). This subgenus was originally erected by Bernhauer (1903) as a subgenus of the genus Priochirus (sensu lato) Sharp, 1887, and this taxonomic treatment was accepted for long time (Bernhauer 1903, Cameron 1930, Greenslade 1971, Naomi 1996). Wu and Zhou (2007) studied the phylogeny of the groups related to the genus Priochirus (sensu lato), and found that the genus was not a monophyletic taxon. Thus, improved taxonomy was proposed: Plastus was raised to a valid genus and the genus Priochirus was retained. Consequently their taxonomic definitions were changed (Wu and Zhou 2007). The genus Plastus Bernhauer, 1903 (sensu Wu and Zhou 2007) includes the following 8 subgenera: Leiochirus Greenslade, 1971, Exochirus Greenslade, 1971, Syncampsochirus Bernhauer, 1903, Eutriacanthus Jakobson, 1908, Plastus s. str., Barychirus Greenslade, 1971, Stigmatochirus Bernhauer, 1903 and Sinumandibulus Wu & Zhou, 2007. These 7 taxa were originally in the genus Priochirus sens. lat. (Greenslade 1971). This emended classification is adopted in this study.

Previously one species, Plastus brachycerus (Kraatz, 1859), was erroneously recorded from China (Wu and Zhou 2007), which is a misidentification of Plastus shanghaiensis sp. n. Five new species are discovered and here described from China: Plastus Plastus amplus Wu & Zhou, sp. n. from Xizang, Plastus Plastus rhombicus Wu & Zhou, sp. n. from Guangxi, Plastus Plastus rhombicus Wu & Zhou, sp. n. from Yunnan, Plastus Plastus shanghaiensis Wu & Zhou, sp. n. from Shanghai, Plastus Plastus tuberculatus Wu & Zhou, sp. n. from Hainan. An earlier confusion (Wu and Zhou 2007) between Plastus brachycerus (Kraatz, 1859) and Plastus shanghaiensis sp. n. is resolved. Our study suggests that the subgenus Plastus s. str. is widely distributed from southwest to east China. This information may shed light on the origin and dispersal of Plastus s. str. in tropical and subtropical Asia.

## Material and methods

All specimens examined were measured using a compound microscope (Leica MZ12). Before dissection the specimens were relaxed in warm water (60°C) for 3–5 hours, then male genitalia were separated from terminal abdominal segment by dissecting needle, macerated in 10% KOH solution, rinsed in distilled water, and preserved in 75% alcohol for consequent observation. Measurements and photographs were taken by using CCD Scientific Cameras (Motic 252A) and digital microscopy software (Motic Images Advanced 3.2 and Multi-Focus 1.0).

The morphological terminology follows mainly that used by Wu and Zhou (2007) and Greenslade (1971, 1972).

The following abbreviations are used in the text: HLhead length (measured from anterior margin of frontal angle to the posterior margin of head capsule); PLpronotum length (measured along medial line of the disc); ELelytron length (measured from the humeral margin to the most distal margin); HWhead width (maximal, excluding eyes); PWpronotum width (maximal); EWelytra width (maximal).

### Depositories

Specimens from this study are deposited in the following collections:

IZ-CASInstitute of Zoology, the Chinese Academy of Sciences, Beijing

SEM-CASShanghai Entomological Museum, the Chinese Academy of Sciences, Shanghai

## Results

### Genus Plastus

Bernhauer, 1903

Plastus Bernhauer 1903: 142, 160 (as subgenus of Priochirus); Wu and Zhou 2007: 81, 85 (valid genus, emended).

#### Type species:

Leptochirus convexus Laporte, 1835, fixed by subsequent designation (Lucas 1920).

#### Subgenus Plastus

Bernhauer, 1903

##### Diagnosis.

This subgenus may be distinguished from the other subgenera by having the head with a pair of distinct lateral teeth; outer lateral teeth on head often present, but never lateral to, nor in the same horizontal plane as main lateral teeth; frontal tooth on anterior margin of frontal impression of head, if present, never single and centrally placed (Bernhauer 1903, Greenslade 1971).

##### Key to the species of subgenus Plastus s. str. from China

**Table:** 

1.	Frontal impression on head rhomboid in shape, anterior margin strongly convex in middle (Fig. 8); epipleural line on elytron absent	Plastus rhombicus sp. n.
–	Frontal impression of head never rhomboid in shape, anterior margin not convex in middle (Figs 6, 7, 9, 10); epipleural line on elytron present	2
2.	Frontal impression about 5 times as wide as long (Fig. 7); central disc of pronotum with two distinct fovae on sides of longitudinal median sulcus	Plastus biconcavus sp. n.
–	Frontal impression at most 4 times as wide as long (Figs 6, 9, 10); central disc of pronotum without fovae	3
3.	Anterior margin of frontal impression of head without frontal tooth at each side (Fig. 9); mentum ventrally with coarse and mutually contiguous ridges, disc rugose without polished region (Fig. 14)	Plastus shanghaiensis sp. n.
–	Anterior margin of frontal impression of head with frontal tooth at each side (Figs 6, 10); mentum ventrally with three transverse ridges, disc between last posterior ridge and basal margin polished (Figs 11, 15)	4
4.	Lateral teeth on head distinctly convergent forward, with dorsal base not distinctly convex (Fig. 6); anterior margin of parameres convex and pointed at base (Fig. 17)	Plastus amplus sp. n.
–	Lateral teeth on head parallel, with dorsal base strongly convex (Fig. 10); anterior margin of parameres rounded at base (Fig. 21)	Plastus tuberculatus sp. n.

.

##### Plastus Plastus amplus

Wu & Zhou sp. n.

urn:lsid:zoobank.org:act:15E05BCE-416D-45FA-8B60-8A2573E1721D

Figs 1 6 11 16, 17 

###### Type material.

Holotype male, Xizang: Motuo, 1370 m, 18.ix.1979, Gentao Jin and Jianyi Wu coll. (SEM-CAS). Paratypes (13 spp.): 2 males and 9 females, same data as for holotype; male, Xizang: Motuo: Kabu, 1100 m, 8.v.1980, Gentao Jin and Jianyi Wu coll. (SEM-CAS); female, Xizang: Motuo, 1000–1200 m, 11.ii.1983, Yinheng Han coll. (IZ-CAS).

###### Description.

####### Measurement.

 Body length: 10.04–11.13 mm; HL: 0.74–0.82 mm; HW: 1.58–1.70 mm; PL: 1.70–1.97 mm; PW: 2.23–2.47 mm; EL: 2.49–2.58 mm; EW: 2.35–2.54 mm.

####### Coloration.

**Figures 1-5:**
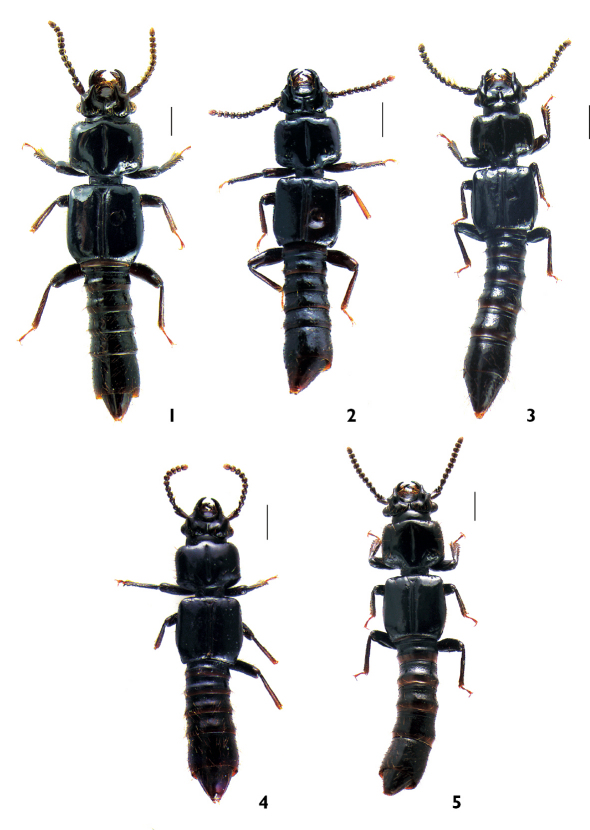
General habitus, dorsal view: **1** Plastus (Plastus) amplus sp. n. **2** Plastus (Plastus) biconcavus sp. n. **3** Plastus (Plastus) rhombicus sp. n. **4** Plastus (Plastus) shanghaiensis sp. n. **5** Plastus (Plastus) tuberculatus sp. n. (scale bar = 1 mm)

**Figures 6-10:**
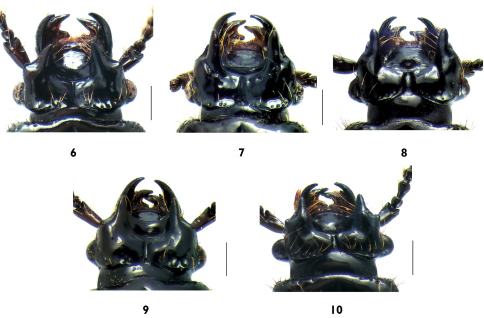
Head, dorsal view: **6** Plastus (Plastus) amplus sp. n. **7** Plastus (Plastus) biconcavus sp. n. **8** Plastus (Plastus) rhombicus sp. n. **9** Plastus (Plastus) shanghaiensis sp. n. **10** Plastus (Plastus) tuberculatus sp. n. (scale bar = 0.5 mm).

 Head black with labrum red-brown, mandibles black with inner side red-brown (Fig. 6). Pronotum and elytra black (Fig. 1). Abdomen black except for 8th segment slightly brown. Antennae brown. Femora black, tibiae black at base and gradually becoming brown apically. Tarsi slightly yellow-brown.

####### Structural attributes.

 Head transverse (Fig. 6), about twice as long as wide, sides slightly concave in middle and slightly convex outward basally; frontal impression strongly transverse, about 3.5 times as wide as long, anterior margin slightly arc-shaped and weakly emarginate in middle with two small granulated frontal teeth on sides, distance between apices of two frontal teeth almost as wide as 2/3 of frontal impression, posterior margin slightly emarginate in middle and slightly obliquely convergent posteriorly; lateral teeth blunt and distinctly convergent anteriorly with apices slightly introflexed, on apical 1/3 of ventral side with distinct subsidiary denticle apically pointed and curved forward, on inner side with row of 5–6 long setae extending onto anterior margin of frontal impression; lateral impression triangularly and widely depressed along almost whole external side of lateral tooth and anterior margin of frontal angle of head, bearing 4–6 short setae; anterior margin of frontal angle of head slightly convex forming small and blunt outer lateral tooth; median sulcus on vertex about twice as long as frontal impression, gradually broadened posteriorly, but abruptly divergent at posterior 1/3, the posterior end of which is almost twice as wide as middle; clypeus short and steeply inclined, anterior margin slightly rounded, baso-laterally with 3–5 long setae; eye glabrous and convex, almost occupying half side of head; vertex moderately convex, in depressed regions near frontal impression with shallow longitudinal depression on each side of median sulcus, surface evenly covered with fine micropunctures, along lateral and postero-lateral margin with some scattered punctures and long setae, at each side of posterior end of median sulcus with 4–7 setiferous punctures concentrated in shallow foveae.

Antennae subgeniculate, antennomere I baculiform and apically slightly depressed to form shallow sulcus on dorsal side, antennomere II smallest, slightly transverse; antennomere III elongate about 1.7 times as long as II; antennomeres IV and V almost quadrate; antennomeres VI–X gradually more transverse apically; antennomere XI elongate, apically rounded, about twice as long as X.

**Figures 11-15:**
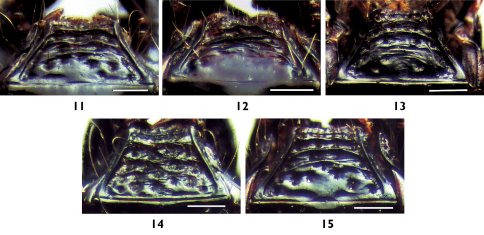
Mentum, ventral view: **11** Plastus (Plastus) amplus sp. n. **12** Plastus (Plastus) biconcavus sp. n. **13** Plastus (Plastus) rhombicus sp. n. **14** Plastus (Plastus) shanghaiensis sp. n. **15** Plastus (Plastus) tuberculatus sp. n. (scale bar = 0.25 m).

Mentum trapeziform (Fig. 11), frontal angle slightly rounded, anterior margin weakly depressed in middle, with small pointed median denticle, ventral surface setose, with three transverse ridges, first distinct and almost straight, second and third slightly wrinkled and indistinct, disc between third ridge and posterior margin polished and with 4 or 5 larger setiferous punctures scattered along base of third ridge.

Pronotum transverse, distinctly wider than head, anterior margin weakly bisinuate, sides almost parallel, median longitudinal sulcus deep and narrow, slightly broadened posteriorly, not reaching either anterior nor posterior margins; lateral marginal area with 30–35 setiferous punctures, punctures on upper half distinctly larger than on lower half, disc polished, with indistinct micropunctures evenly distributed.

Protibiae externally furnished with 11–15 denticles, which gradually become shorter basally.

Elytra almost quadrate, epipleural line complete, setiferous punctures on lateral marginal side sparser and less distinct than those on sides of pronotum.

Abdomen cylindrical, along anterior and posterior margin of III–VI segments respectively with row of setae, but VI segment distinctly with additional median row of setae extending to central disc, segments VII and VIII densely setose, disc of III–VI segment densely punctured except for transverse glabrous region near to posterior margin, basal distinctly denser than apical, on disc of VII and VIII, centrally with longitudinal glabrous region, narrow and extending to basal region.

**Figures 16, 17:**
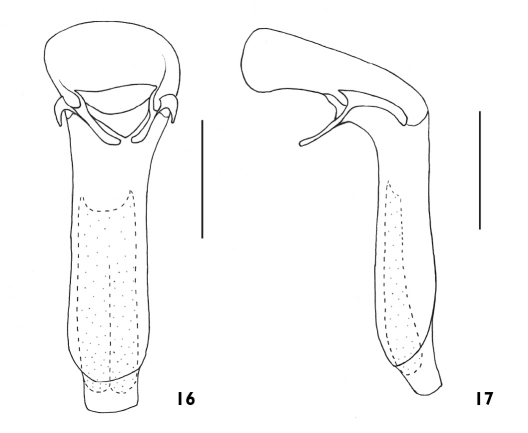
Aedeagus of Plastus (Plastus) amplus sp. n.: **16** ventral view **17** lateral view (scale bar = 0.5 mm).

Aedeagus submembranous (Figs 16, 17), basal part of median lobe slightly bulbous and strongly curved behind basal orifice, almost L-shaped in lateral view; posterior part baculiform, slightly narrower than basal bulbous part, sides almost parallel, distinctly sclerotised on both ventral and lateral sides, with apex membranous and protruding; parameres elongate and weakly curved with apices not extending beyond apical level of basal protruding part, anterior margin strongly convex and pointed near base; basal part protruding ventrally, but not connecting to each other below basal orifice.

###### Remarks.

This new species is similar to Plastus Plastus brachycerus (Kraatz, 1859), but can be distinguished from the latter by more transverse frontal impression, anteriorly convergent lateral teeth and small outer lateral teeth on anterior margin of frontal angle of head (Fig. 6).

###### Distribution.

Only known from type locality in Xizang, elevations of 1000–1370 m.

###### Etymology.

The species name is derived from Latin word ‘amplus’ (broad) to indicate distinctly transverse frontal impression of head.

##### Plastus Plastus biconcavus

Wu & Zhou sp. n.

urn:lsid:zoobank.org:act:F7BDE46E-673A-462B-BE3D-9484B4B89FC1

Figs 2 7 12 

###### Type material.

Holotype male, Guangxi: Longsheng: Neicujiang, 840 m, 7.vi.1963, Shuyong Wang coll. (IZ-CAS).

###### Description.

####### Measurement.

 Body length: 9.84 mm. HL: 0.71 mm; HW: 1.48 mm; PL: 1.56 mm; PW: 2.07 mm; EL: 2.18 mm; EW: 2.09 mm.

####### Coloration.

 Head black with labrum red-brown, mandibles black with inner side reddish brown (Fig. 7). Pronotum and elytra slightly dark brown (Fig. 2). Abdomen black except for 8th segment slightly rufous. Antennae dark red brown. Femora and tibiae slightly dark red brown. Tarsi brown.

####### Structural attributes.

 Head transverse (Fig. 7), twice as long as wide, sides slightly concave in middle; frontal impression strongly transverse, about 5 times as wide as median length, anterior margin almost straight in middle, posterior margin slightly rounded posteriorly; lateral teeth blunt and straightly projecting, middle of ventral side with bluntly convex subsidiary denticle, on inner side with row of 4–6 long setae extending onto anterior margin of frontal impression; lateral impression narrow, along posterior half of external side of lateral tooth, almost not reaching anterior margin of frontal angle of head, bearing 3 or 4 short setae; anterior margin of frontal angle of head rounded, without tooth; median sulcus on vertex about 3 times as long as median length of frontal impression, gradually broadened posteriorly, but abruptly divergent at posterior 1/4, posterior end of which almost twice as wide as middle; clypeus steeply inclined and rounded anteriorly, with shallow depression behind anterior margin, baso-laterally with 2 or 3 long setae; eye glabrous and convex, almost occupying half side of head; vertex broadly convex, between frontal angle and base of lateral tooth with luniform depression, in depressed region near posterior margin of frontal impression with two distinct punctures in line on each side of median sulcus, surface polished and evenly covered with fine micropunctures, along lateral and postero-lateral margin with some scattered punctures and long setae, at each side of posterior end of median sulcus without distinct fovea.

Antennae subgeniculate, antennomere I baculiform and apically slightly depressed, antennomere II smallest, slightly transverse; antennomere III elongate about 2 times as long as II; antennomeres IV quadrate; antennomeres V–X gradually more transverse apically; antennomere XI elongate, apically rounded, about 2 times as long as X.

Mentum trapeziform (Fig. 12), frontal angle slightly rounded, anterior margin weakly depressed in middle, with small indistinct median denticle, ventral surface with three transverse and waved ridges, but third indistinct and vague, space between ridges setose and rugose, but disc between last posterior ridge and basal margin glabrous and polished, with two large setiferous punctures on sides.

Pronotum transverse, distinctly wider than head, anterior margin weakly bisinuate, sides almost parallel, median longitudinal sulcus deep, broadest in middle and gradually narrowed anteriorly and posteriorly, not reaching either anterior or posterior margins; lateral marginal area with 21–23 setiferous punctures, punctures on upper half not distinctly larger than on lower, disc polished, surface evenly with fine micropuncture scattered, centrally with two distinct fovea on sides of longitudinal sulcus.

Protibiae externally furnished with 13 or 14 denticles, which gradually become shorter basally.

Elytra slightly longer than wide, epipleural line complete, setiferous punctures on lateral marginal side sparser and less distinct than those on side of pronotum.

Abdomen cylindrical, along anterior and posterior margin of III–VI segments respectively with row of setae, segments VII and VIII densely setose, disc of III–VI segment densely punctured, except for broad and transverse glabrous region near posterior margin, basal denser than apical, central disc of VII evenly punctured, VIII centrally with narrow longitudinal glabrous region.

###### Remarks.

This new species can easily be distinguished from other members of the subgenus Plastus s. str. by its strongly transverse frontal impression (about 5 times as wide as long) and two distinct foveae on sides of pronotal disc. Plastus Plastus taprobanus (Cameron, 1930) also has strongly transverse frontal impression and distinct fovea on either side of pronotum, but its lateral teeth are distinctly shorter than those in this new species.

###### Distribution.

Known from the type locality in Guangxi, elevation of 840 m.

###### Etymology.

The species name is derived from Latin words ‘bi-’ (double) and ‘concavus’ (concave) to indicate two distinct fovae on pronotum of this species.

##### Plastus Plastus rhombicus

Wu & Zhou sp. n.

urn:lsid:zoobank.org:act:91C17665-3964-4AD5-AA9C-B83CE75140F4

Figs 3 8 13 18 19 

###### Type material.

Holotype male, Yunnan: Tengchong: Jietou (25°41.820'N 098°40.835'E), 1865 m, 14.v.2006, H.B. Liang coll. (IZ-CAS). Paratypes (2 spp.): 2 females, same data as for holotype (IZ-CAS).

###### Description.

####### Measurement.

 Body length: 10.43–11.78 mm. HL: 0.56–0.70 mm; HW: 1.43–1.52 mm; PL: 1.46–1.67 mm; PW: 1.99–2.22 mm; EL: 2.01–2.21 mm; EW: 2.09–2.25 mm.

####### Coloration.

 Head black with labrum red-brown (Fig. 8), mandibles black with inner side red-brown. Pronotum, elytra and abdomen black (Fig. 3). Antennae dark brown. Femora black, tibiae black at base and gradually become red-brown apically. Tarsi brown.

####### Structural attributes.

 Head (Fig. 8) transverse, about twice as long as wide, sides slightly divergent anteriorly; frontal impression deep and rhomboid, with anterior margin medially convex and slightly turned up, posterior margin slightly convergent posteriorly, about 3 times as wide as long; lateral teeth blunt with apices slightly divergent forward, on apical 1/3 of ventral side with triangularly convex subsidiary denticle (in front of which, additional small denticle present in one examined female specimen), on inner side with row of 5–7 long setae, not extending onto anterior margin of frontal impression; along external side of lateral tooth, lateral impression very short and narrow, not extending to front angle of head, bearing 2–4 short setae, anterior margin of frontal angle of head rounded; median sulcus on vertex almost twice as long as median length of frontal impression, gradually broadened posteriorly, the posterior end of which is almost 2 times as wide as middle; clypeus elongate and rounded anteriorly, with shallow depression behind anterior margin, along lateral side with 4 or 5 long setae scattered; eye glabrous and convex, occupying about 3/5 of side of head; vertex strongly convex near frontal angle, central disc polished and covered with fine micropunctures, along lateral and postero-lateral margin with some scattered punctures and long setae, at each side of posterior end of median sulcus with indistinct fovea bearing 2 or 3 long setae.

Antennae subgeniculate, antennomere I baculiform and apically slightly depressed to form shallow sulcus on dorsal side, antennomere II smallest, slightly transverse; antennomere III elongate about 1.5 times as long as II; antennomeres IV–VI slightly quadrate; VII–X gradually more transverse apically; antennomere XI elongate, apically rounded, about 1.5 times as long as X. Mentum trapeziform (Fig. 13), frontal angle slightly rounded, anterior margin weakly depressed in middle, with indistinct median denticle, ventral surface setose, with three wrinkled ridges, last posterior one less distinct than anterior two, disc surface with 4 large setiferous punctures along posterior margin, space between those punctures slightly rugose.

Pronotum transverse (Fig. 3), distinctly wider than head, anterior margin weakly bisinuate, sides rounded and slightly convergent anteriorly, median longitudinal sulcus deep and narrow, slightly broadened posteriorly, not reaching either anterior or posterior margins; lateral marginal area with 24–37 setiferous punctures, punctures on upper half slightly larger than those on lower half, disc polished, with fine micropuncture evenly distributed.

Protibiae externally furnished with 11–14 denticles, which gradually become shorter basally.

Elytra almost quadrate, epipleural line absent, setiferous punctures on lateral marginal side sparser and less distinct than those on side of pronotum.

Abdomen cylindrical, along anterior and posterior margin of III–VI segments respectively with row of setae, disc of each segments densely punctured, except for broad glabrous region near posterior margin, basal distinctly denser than apical, segments VII and VIII densely setose and punctured, with longitudinal glabrous region in central.

**Figures 18, 19:**
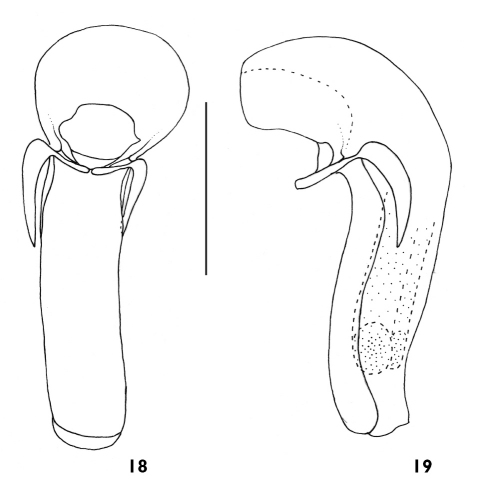
Aedeagus of Plastus (Plastus) rhombicus sp. n.: **18** ventral view **19** lateral view (scale bar = 0.5 mm).

Male: aedeagus almost membranous (Figs 18, 19), basal part of median lobe strongly bulbous and curved behind basal orifice; posterior part baculiform, distinctly narrower than basal part, sides almost parallel, only ventral side sclerotised; parameres elongate and strongly curved with apices far below level of basal protruding apices, anterior margin near base rounded; basal parts protruding ventrally, apically weakly separated below basal orifice.

###### Remarks

. This species can be distinguished from other members of subgenus Plastus s. str. by the rhomboid shape of frontal impression of head (Fig. 8). The anterior margin of frontal impression in this species is convex upward in middle. This structure is slightly similar in position to the central tooth in the subgenus Eutriacanthus, but never pointed and protruding anteriorly as the single central tooth in the latter. Thus, this new species has a clinal morphological characteristics between subgenera Eutriacanthus and Plastus s. str.

###### Distribution.

Known from the type locality in Yunnan, elevation of 1865 m.

###### Etymology.

The species name derived from a Latin word ‘rhombicus’ (rhombic) to indicate the shape of its frontal impression.

##### Plastus Plastus shanghaiensis

Wu & Zhou sp. n.

urn:lsid:zoobank.org:act:DBDA5500-9257-452F-9C87-8C92A0B2DD05

Figs 4 9 14 

###### Type material.

Holotype female,Zi Ka Wei (Shanghai: Xujiahui), 4.v.1939, collector unknown (IZ-CAS).

###### Description.

####### Measurement.

 Body length: 9.75 mm. HL: 0.72 mm; HW: 1.42 mm; PL: 1.63 mm; PW: 2.02 mm; EL: 2.05 mm; EW: 2.14 mm.

####### Coloration.

 Head black with labrum red-brown (Fig. 9), mandibles black with inner side red-brown. Pronotum and elytra black (Fig. 4). Abdomen black with posterior margin of VII and whole VIII segment rufous. Antennae brown. Femora black, tibiae black at base and gradually become red-brown apically. Tarsi red-brown.

####### Structural attributes.

 Head (Fig. 9) transverse, twice as long as wide, sides almost parallel, slightly concave in middle; frontal impression transverse, about 2 times as wide as median length, anterior margin almost semicircular, posterior margin rounded; lateral teeth straight and blunt, ventral side medially with weakly convex subsidiary denticle, on inner side with row of 2 or 3 long setae extending onto lateral anterior margin of frontal impression; lateral impression narrowly depressed at outer base of lateral tooth, bearing 2 or 3 short setae, anterior margin of frontal angle of head weakly convex and forming small granulated tooth; median sulcus on vertex about 1.5 times as long as median length of frontal impression, gradually broadened posteriorly, but abruptly divergent at posterior 1/4, the posterior end of which is almost 2.5 times as wide as middle; clypeus short and steeply inclined, anterior margin moderately rounded, with 3–5 long setae scattered along lateral side; eye glabrous and convex, occupying about 3/5 of side of head; vertex moderately convex, gradually inclining in regions near posterior margin of frontal impression, without distinct depression on each side of median sulcus, surface almost polished and covered with fine micropunctures, along lateral and postero-lateral margin with some scattered punctures and long setae, at each side of posterior end of median sulcus with indistinct fovea bearing 2 or 3 setiferous punctures.

Antennae subgeniculate, antennomere I baculiform and apically slightly depressed to form shallow sulcus on dorsal side, antennomere II smallest, slightly transverse; antennomere III elongate about 2 times as long as II; antennomeres IV–V slightly quadrate; VI–X gradually transverse; antennomere XI elongate, apically rounded, about 1.8 times as long as X.

Mentum trapeziform (Fig. 14), frontal angle slightly rounded, anterior margin weakly depressed in middle, with indistinct median denticle, ventral surface with coarse and mutually contiguous ridges, behind which disc strongly rugose and setose.

Pronotum transverse (Fig. 4), distinctly wider than head, anterior margin slightly bisinuate, sides almost parallel, but slightly convex at anterior angle, median longitudinal sulcus deep and narrow, not distinctly broadened posteriorly, not reaching either anterior or posterior margins; lateral marginal area with 17–20 setiferous punctures, punctures on upper half not larger than on those lower half, disc polished, with fine micropuncture evenly distributed, in central with two indistinct punctures at sides of sulcus.

Protibiae externally furnished with 10 denticles, which gradually become shorter basally.

Elytra almost quadrate, epipleural line complete, setiferous punctures on lateral marginal side sparser and less distinct than those on side of pronotum.

Abdomen cylindrical, along anterior and posterior margin of III–VI segments respectively with row of setae, but VI segment with additional median row of setae, segments VII and VIII densely setose, disc of III–VI segments densely punctured, without distinct glabrous region near posterior margin, basal distinctly denser than apical, VII punctured, centrally without longitudinal glabrous region, glabrous region on VIII short and not extending to basal half.

###### Remarks.

Wu and Zhou (2007) misidentified this species as Plastus Plastus brachycerus (Kraatz, 1859) (tables 1, 2; figures 1–4; and locality error in Wu and Zhou 2007). Plastus (Plastus) shanghaiensis sp. n. is closely allied to Plastus Plastus brachycerus in the shape of lateral teeth and frontal impression, but can be distinguished from the latter by more depressed lateral impression at the outer base of lateral tooth, and anterior margin of frontal angle of head is furnished with a small granulate tooth.

###### Distribution.

Known from type locality in Shanghai.

###### Etymology.

The specific name ‘shanghaiensis’ is derived from the name of the type locality, Shanghai.

##### Plastus Plastus tuberculatus

Wu & Zhou sp. n.

urn:lsid:zoobank.org:act:A4D0B921-8BB3-4802-B6E7-06AF37BEC6E9

Figs 5 10 15 20, 21 

###### Type material.

Holotype male, Hainan: Diaoluoshan, 14.i.1985, Gentao Jin and Zurao Liu coll. (SEM-CAS). Paratypes (18 spp.): 11 males, 6 females, same data as for holotype (SEM-CAS); male, Hainan: Limushan: Sanquling, 30.xi.2007, 830 m, Zhuo Yang coll. (IZ-CAS).

###### Description.

####### Measurement.

 Body length: 9.80–10.35 mm. HL: 0.61–0.66 mm; HW: 1.47–1.65 mm; PL: 1.63–1.81 mm; PW: 2.15–2.29 mm; EL: 2.14–2.32 mm; EW: 2.17–2.22 mm.

####### Coloration.

 Head black with labrum red-brown (Fig. 10), mandibles black with inner side red-brown. Pronotum and elytra black (Fig. 5). Abdomen black, except for 8th segment slightly rufous. Antennae brown. Femora black, tibiae slightly red-brown. Tarsi slightly yellow-brown.

####### Structural attributes.

 Head (Fig. 10) transverse, twice as long as wide, lateral sides slightly concave in middle; frontal impression strongly transverse, about 4 times as wide as long, anterior margin deeply emarginate in middle, with two triangularly convex and pointed frontal teeth at sides, distance between apices of two frontal teeth almost as wide as 3/5 of frontal impression, posterior margin slightly obliquely convergent backwards; lateral teeth straight and blunt, on apical 1/3 of ventral side with distinct pointed subsidiary denticle, on inner side with row of 5–8 long setae extending onto outer base of frontal tooth; lateral impression triangularly and widely depressed at outer base of lateral tooth, bearing 5 or 7 short setae; anterior margin of frontal angle of head slightly convex and depressed inside, forming blunt outer lateral tooth; median sulcus about 1.5 times as long as median length of frontal impression, distinctly divergent posteriorly, almost triangular, the posterior end of which is almost two times as wide as middle; clypeus short and steeply inclined, anterior margin moderately rounded, with 3–5 long setae scattered along lateral side; eye glabrous and strongly convex, occupying about 3/5 of side of head; vertex strongly and tuberculately convex at dorsal base of lateral tooth, gradually inclining in regions near posterior margin of frontal impression, without distinct depression on each side of median sulcus, surface almost polished and covered with fine micropunctures, along lateral and postero-lateral margin with some scattered punctures and long setae, at each side of posterior end of median sulcus with distinct fovea bearing 5–7 setiferous punctures.

Antennae subgeniculate, antennomere I baculiform and apically slightly depressed to form shallow sulcus on dorsal side, antennomere II smallest, slightly transverse; antennomere III elongate about 2 times as long as II; antennomeres IV–X gradually transverse, X about 2.5 times as wide as long; antennomere XI elongate, apically rounded, about 2.5 times as long as X.

Mentum trapeziform (Fig. 15), frontal angle slightly rounded, anterior margin weakly depressed in middle, with indistinct median denticle, ventral surface with three transverse and slightly waved ridges, disc between last posterior ridge and basal margin moderately polished, with 5 or 6 large setiferous punctures.

Pronotum transverse (Fig. 5), distinctly wider than head, anterior margin very weakly bisinuate, sides slightly convergent anteriorly at anterior 1/4; median longitudinal sulcus deep and narrow, slightly broadened at posterior end, not reaching either anterior or posterior margins; lateral marginal area with 30–40 setiferous punctures, punctures on upper half distinctly larger than on those lower half, disc polished, with fine micropuncture evenly distributed.

Protibiae externally furnished with 11–13 denticles, which gradually become shorter basally.

Elytra almost quadrate, epipleural line complete, setiferous punctures on lateral marginal side sparser and less distinct than those on side of pronotum.

Abdomen cylindrical, along anterior and posterior margin of III–VI segments respectively with row of setae, but VI segment with additional median row of setae, segments VII and VIII densely setose, disc of III–VI segment densely punctured, basal distinctly denser than apical, but near posterior margin with small glabrous region in middle, on disc of VII and VIII, glabrous region narrow and extending to basal region.

**Figures 20-21:**
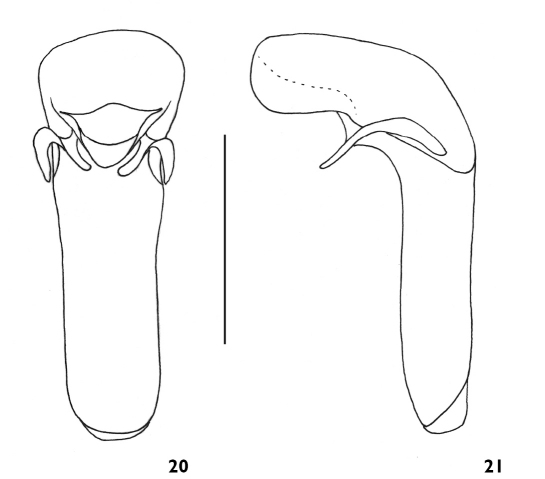
Aedeagus of Plastus (Plastus) tuberculatus sp. n.: **20** ventral view **21** lateral view (scale bar = 0.5 mm).

Male aedeagus almost membranous (Figs 20, 21), basal part of median lobe bulbous and curved behind basal orifice; posterior part baculiform, almost as broad as basal part, sides slightly constricted in middle, distinctly sclerotised on both ventral and lateral sides, with membranous and protruding apex; parameres elongate and weakly curved with apices not extending beyond level of basal protruding apices, slightly broadened near base with anterior margin rounded; basal parts protruding ventrally, apically widely separated below basal orifice.

###### Remarks.

This species is allied to Plastus Plastus kimurai (Naomi, 1996) in the shape of lateral and outer lateral teeth on head, but can be distinguished from the latter by following features: anterior margin of frontal impression with two distinct frontal teeth, dorsal base of lateral teeth tuberculately convex and median sulcus on head distinctly broadened posteriorly (Fig. 10).

###### Distribution.

Known from type locality in Hainan, elevation about 800 m.

###### Etymology.

The species name is derived from Latin word ‘tuberculatus’ (tuberculate) to indicate vertex tuberculately convex at base of lateral teeth.

## Supplementary Material

XML Treatment for Plastus
