# Corrigenda: Wu J, Zhou H-Z (2010) Taxonomic study of subgenus *Plastus* s. str. (Coleoptera, Staphylinidae, Osoriinae) in China, with descriptions of five new species

**DOI:** 10.3897/zookeys.67.706

**Published:** 2010-11-10

**Authors:** Jie Wu, Hong-Zhang Zhou

**Affiliations:** 1 Key Laboratory of Zoological Systematics and Evolution, Institute of Zoology, Chinese Academy of Sciences, 1 Beichenxi Rd., Chaoyang District, Beijing 100101, P. R. China; 2 Shanghai Entomological Museum, Chinese Academy of Sciences, 300 Fenglin Rd., Xuhui District, Shanghai 200032, P. R. China

In the paper of Wu and Zhou (Zookeys 51: 17–32, doi: 10.3897/zookeys.51.457), the below text:

Jie Wu 1,†, Hong-Zhang Zhou 2,‡

1	Key Laboratory of Zoological Systematics and Evolution, Institute of Zoology, Chinese Academy of Sciences, 1 Beichenxi Rd., Chaoyang District, Beijing 100101, P. R. China

2	Shanghai Entomological Museum, Chinese Academy of Sciences, 300 Fenglin Rd., Xuhui District, Shanghai 200032, P. R. China

should read:

Jie Wu 1,2,†, Hong-Zhang Zhou 1,‡

1	Key Laboratory of Zoological Systematics and Evolution, Institute of Zoology, Chinese Academy of Sciences, 1 Beichenxi Rd., Chaoyang District, Beijing 100101, P. R. China

2	Shanghai Entomological Museum, Chinese Academy of Sciences, 300 Fenglin Rd., Xuhui District, Shanghai 200032, P. R. China

